# Online Health Information Seeking and eHealth Literacy Among Spanish Language–Dominant Latino Adults Receiving Care in a Community Clinic: Secondary Analysis of Pilot Randomized Controlled Trial Data

**DOI:** 10.2196/37687

**Published:** 2022-10-13

**Authors:** Enmanuel A Chavarria, Shannon M Christy, Han Feng, Hongyu Miao, Rania Abdulla, Liliana Gutierrez, Diana Lopez, Julian Sanchez, Clement K Gwede, Cathy D Meade

**Affiliations:** 1 Department of Behavioral, Social, and Health Education Sciences Rollins School of Public Health Emory University Atlanta, GA United States; 2 Cancer Prevention and Control Research Program Winship Cancer Institute Emory University Atlanta, GA United States; 3 Department of Health Outcomes and Behavior Division of Population Science H. Lee Moffitt Cancer Center and Research Institute Tampa, FL United States; 4 Department of Oncologic Sciences Morsani College of Medicine University of South Florida Tampa, FL United States; 5 Department of Gastrointestinal Oncology H. Lee Moffitt Cancer Center and Research Institute Tampa, FL United States; 6 School of Medicine Tulane University New Orleans, LA United States; 7 College of Nursing Florida State University Tallahassee, FL United States; 8 Non-Therapeutic Research Office H. Lee Moffitt Cancer Center and Research Institute Tampa, FL United States; 9 Evergreen Health Kirkland, WA United States; 10 Suncoast Community Health Centers Brandon, FL United States

**Keywords:** eHealth literacy, online health information seeking, medically underserved, Hispanic, Latino, Spanish language–dominant, health communications, colorectal cancer screening

## Abstract

**Background:**

eHealth literacy is the ability to seek, obtain, and decipher online health information (OHI) for health and disease management. Rapid developments in eHealth (eg, health care services and online information) place increased demands on patients to have high eHealth literacy levels. Yet, greater emphasis on eHealth may disproportionately affect groups with limited eHealth literacy. Cultural background, language, and eHealth literacy are influential considerations affecting health care and information access, health care use, and successful eHealth resource use, and they may influence OHI seeking for behavioral change toward cancer prevention.

**Objective:**

This study aimed to characterize the extent of OHI seeking and eHealth literacy among Spanish-dominant (SD) Latino adults aged 50 to 75 years. Further, we aimed to examine potential associations between sociodemographic characteristics, Preventive Health Model (PHM) constructs, OHI-seeking behaviors, and eHealth literacy, separately.

**Methods:**

Participants (N=76) self-identified as Latino, were enrolled in a colorectal cancer (CRC) screening intervention, were aged 50 to 75 years, were at average risk for CRC, were not up to date with CRC screening, and preferred receiving health information in Spanish. We describe participants’ sociodemographic characteristics, PHM constructs, OHI-seeking behaviors, and eHealth literacy—among those seeking OHI—assessed at enrollment. Descriptive analyses were first performed for all variables. Next, primary univariate logistic analyses explored possible associations with OHI seeking. Finally, using data from those seeking OHI, exploratory univariate analyses sought possible associations with eHealth literacy.

**Results:**

A majority (51/76, 67%) of the participants were female, 62% (47/76) reported not having graduated high school, and 41% (31/76) reported being unemployed or having an annual income of less than US $10,000. Additionally, 75% (57/76) of the participants reported not having health insurance. In total, 71% (54/76) of the participants reported not having sought OHI for themselves or others. Univariate logistic regression suggested that higher educational attainment was significantly associated with an increased likelihood of having sought OHI (odds ratio 17.4, 95% CI 2.0-150.7; *P*=.009). Among those seeking OHI (22/76, 29%), 27% (6/22) were at risk of having low eHealth literacy based on an eHealth Literacy Scale score of less than 26. Among OHI seekers (22/76, 29%), an examination of associations found that higher eHealth literacy was associated with greater self-efficacy for screening with the fecal immunochemical test (*β*=1.20, 95% CI 0.14-2.26; *P*=.02).

**Conclusions:**

Most SD Latino participants had not sought OHI for themselves or others (eg, family or friends), thus potentially limiting access to beneficial online resources. Preliminary findings convey that higher eHealth literacy occurs among those with higher self-efficacy for CRC screening. Findings inform areas of focus for future larger-scale investigations, including further exploration of reasons for not seeking OHI among SD Latino adults and an in-depth look at eHealth literacy and cancer screening behaviors.

**Trial Registration:**

ClinicalTrials.gov NCT03078361; https://clinicaltrials.gov/ct2/show/NCT03078361

## Introduction

Today, an ever-increasing quantity of health information can be accessed online [1,2]. Accordingly, online health information (OHI) is now becoming commonplace in health care interactions and health education for patients and caregivers [3-5]. Such developments place an increased demand on patients to be able to seek, obtain, and decipher OHI for health and disease management [6-9]. eHealth tools provide little value if the intended users require added training and skills to effectively engage these resources. These skills are termed “eHealth literacy” and comprise a multifaceted dynamic construct, including previous and current technology use, demographic and cognitive status, and health-related quality of life (HRQOL) [9,10].

A growing evidence base now attests to the promise of eHealth for promoting positive health behavior change, self-efficacy, and knowledge acquisition [11-13].

In recognition of the importance of health information access, the US Department of Health and Human Services set objectives in 2010 for health communication (HC) and health information technology (HIT) as part of the Healthy People initiative. Today, Healthy People 2030 offers HC and HIT objectives aimed at enhancing the use of OHI in public health, including the following: (1) “Increase the proportion of people who can view, download, and send their electronic health information” [14] and (2) “Increase the proportion of people who say their online medical record is easy to understand” [15].

Access to OHI is not uniformly distributed throughout the population, which may exacerbate disparities in health and health care [16,17].

Among Latino adults, the fastest growing demographic group in the United States, 72% overall are using the internet [18]. Yet, among Spanish-dominant (SD) Latino adults aged 50 to 64 years, internet use drops to 67%, and only 42% of those aged 65 years and older use the internet [19]. Since disparities exists, it is necessary to understand the current OHI and eHealth literacy levels of SD Latino adults aged 50 to 75 years, yet there is insufficient knowledge in this area.

Further, among Latino adults, cancer continues to be the leading cause of death, accounting for 21% of overall deaths [8,20,21]. Specifically, colorectal cancer (CRC) is the third leading cause of cancer deaths among Latino adults [21].

The Preventive Health Model (PHM) is a conceptual framework that aims to explain how health beliefs are related to CRC screening [22]. Specifically, among the CRC screening literature, 26 items measure seven PHM constructs. The PHM constructs include salience and coherence, perceived susceptibility, self-efficacy, response efficacy, cancer worry, social influence, and religious beliefs [23]. These are assessed using a 5-point Likert scale, ranging from 1 (strongly disagree) to 5 (strongly agree) [22,24-26]. Reliability and validity for these subscales have been demonstrated previously [22,24-26]. The salience and coherence subscale measures one’s belief that CRC screening is important and makes sense in one’s life. The perceived susceptibility subscale assesses one’s perceived risk of being diagnosed with CRC. The self-efficacy subscale measures the belief that one could complete the steps necessary for fecal immunochemical test (FIT) collection. The response efficacy subscale measures the belief that CRC screening is beneficial for early detection and prevention of CRC. The cancer worry subscale measures the degree to which one is worried about having an abnormal CRC screening result. The social influence subscale assesses the perception that important others (eg, family members, friends, and one’s health care provider) would want the individual to complete CRC screening. The religious beliefs subscale assesses the degree to which one relies on one’s religious beliefs to make health decisions. Thus, in relating a need to understand current OHI and eHealth literacy levels of SD Latino adults aged 50 to 75 years, it is also of interest to explore how these may relate to PHM constructs in this understudied population. Prior studies suggest that sociodemographic and PHM constructs, such as higher social influence and religious beliefs, were associated with lower health literacy among English-preferring individuals [27,28]. By contrast, lower cancer worry and lower religious beliefs [29] were associated with adequate health literacy to understand written health information, and higher educational attainment was significantly associated with adequate health literacy in completing health forms among SD Latino adults aged 50 to 75 years [29]. However, less is known about how sociodemographic variables and PHM constructs might influence OHI seeking or eHealth literacy among SD Latino adults.

Thus, the aims of this study were to (1) describe the prevalence of OHI seeking and eHealth literacy and (2) examine preliminary associations between sociodemographic characteristics, PHM constructs, OHI seeking, and eHealth literacy, separately, among SD Latino adults.

To achieve our study aims, we posed the following four research questions:

1. Research question 1 (RQ1): To what extent do SD Latino adults use the internet to locate OHI?
2. Research question 2 (RQ2): How are self-reported levels of eHealth literacy described among SD Latino adults who seek OHI?
3. Research question 3 (RQ3): What sociodemographic characteristics and PHM constructs are associated with OHI seeking by SD Latino adults?
4. Research question 4 (RQ4): What sociodemographic characteristics and PHM constructs are preliminarily associated with eHealth literacy among SD Latino adults who seek OHI?

Taken together, this study examines OHI and eHealth literacy among a diverse understudied population (ie, SD Latino adults). Further, the study provides a preliminary look at possible associations among sociodemographic characteristics, PHM constructs, OHI seeking, and eHealth literacy through an innovative examination within the literature.

## Methods

### Overview

Data for this report were collected as part of a larger pilot randomized controlled trial—Latino Colorectal Cancer Awareness, Research, Education, and Screening (Latino CARES)—that promoted CRC screening by providing education and a FIT. The pilot study aimed to evaluate the feasibility and impact of a Spanish-language, low-literacy, culturally targeted intervention (ie, a photonovella and DVD) plus FIT compared with a standard Spanish-language booklet developed by the Centers for Disease Control and Prevention plus FIT. Of note, recruitment took place at two participating clinic sites that are part of a Federally Qualified Health Center (FQHC) in Southwest Florida. The FQHC sites are centrally located in agricultural farmworker communities and annually serve a large number (ie, approximately 5000) of medically underserved patients aged 50 to 75 years, a majority of whom are of Latino origin from diverse nationalities and include farmworker populations [30]. Detailed methods are provided in prior publications that communicate the results of the main outcomes of the Latino CARES study [29-31].

Participant consent was obtained prior to baseline interview and randomization. Eligible participants (1) were receiving care at two participating FQHC clinic locations; (2) were between the ages of 50 and 75 years; (3) self-identified as Latino; (4) were able to read, speak, and understand Spanish; (5) preferred to receive health information in Spanish; (6) were currently not up to date per CRC screening guidelines (ie, had never screened or previously screened but were now overdue); and (7) were at average risk for CRC.

### Ethical Considerations

The University of South Florida Institutional Review Board approved the study (approval No. MCC-17665) prior to participant enrollment. Study procedures were conducted in accordance with the Declaration of Helsinki. During enrollment, participants provided informed consent to be included in the study.

### Measures

Study assessments consisted of validated measures that were translated into Spanish and refined for cultural relevance using the following established procedures. Our study team included three bilingual (ie, fluent in English and Spanish) researchers and a bilingual (ie, fluent in English and Spanish) community advisory board. Applying the Brislin method [32], measures were first translated into Spanish by a bilingual study coordinator and then back-translated by a second bilingual study team member. Any discrepancies were arbitrated by a third bilingual study team member. Items were pretested among community members using learner verification methodology [33,34]; they were further refined in consultation with the community advisory board for cultural relevance and comprehension. Baseline items were administered by bilingual (ie, English and Spanish) study coordinators at the time of interview. To minimize literacy issues, all questions were read aloud for all participants.

### Online Health Information Seeking

A single item, gathered from the Pew Research Center [8] and used in previous OHI behavioral studies among Latino adults [4,35], assessed OHI-seeking usage: “Have you previously personally searched for health information on the Internet/Online for yourself or for others? For example, [have you] sought on Google/Yahoo information on high blood pressure, healthy recipes, or efficient exercises?” Response options were yes or no.

### eHealth Literacy

eHealth literacy was assessed only among participants who reported engaging in OHI seeking. Thus, the number of participants for whom eHealth literacy was assessed was lower than that for those responding to the other measures. The Spanish-translated eHealth Literacy Scale (eHEALS) [9,36] was administered to assess eHealth literacy, including knowledge, comfort, and perceived skills at finding, evaluating, and applying OHI to health problems. The eHEALS [9] comprises eight items scored on a 5-point Likert scale, ranging from 1 (strongly disagree) to 5 (strongly agree), and aims to reflect the individuals’ own perceptions of their knowledge and skills at using eHealth information [9,37]. The final result is the sum of all items and ranges from 8 to 40, with higher scores reflecting a higher level of eHealth literacy. The validity and reliability of the eHEALS has been demonstrated across various health conditions [38,39], ages [40-42], and languages [43], including Spanish [36]. Following other studies with similar target populations, the cutoff for high eHealth literacy was set at 26 [10,38,44-48]. Thus, in maintaining consistency in terminology with the literature, this study defined high self-perceived eHealth literacy as an eHEALS score equal to or greater than 26 out of 40, and low self-perceived eHealth literacy was defined as an eHEALS score of less than 26 [10,38,44-48].

### Sociodemographic Characteristics

Sociodemographic variables assessed included age, gender, race and ethnicity, marital status, education, health insurance status, employment status, and income. Additionally, as aggregated data in prior literature masked substantial heterogeneity within the Latino population, we assessed parental foreign-born status and participant foreign-born status; if foreign-born, the country of origin and years lived in the United States were also assessed [30].

### Preventive Health Model Variables

Seven constructs of the PHM were assessed in this study using 26 total items referenced from prior CRC studies [24,25,27,28,30,49-52]. For each item assessing PHM constructs, response options were based on a 5-point Likert scale, ranging from 1 (strongly disagree) to 5 (strongly agree). Four items assessed salience and coherence, or the perception that performing a health behavior is consistent with their beliefs about how to protect and maintain health [24-26,29,30,49,50]. Three items assessed perceived susceptibility, or one’s perceived personal risk for developing CRC or colon polyps [24,25,30,49]. Two items assessed cancer worry, or one’s concern that completing CRC screening will reveal a health concern [24,26,29,30,49,50,53].

Two items measured response efficacy, or the belief that adopting a behavior will be effective in reducing disease threat [24,29,30,49,50]. Four items measured social influence, or the influence of family members and doctors or health professionals on an individual’s willingness to comply with CRC screening [24,26,29,30,49,50]. Five items assessed religiosity, or the extent to which religious beliefs might influence medical decision–making, such as CRC screening [29,30,51,54]. Six items measured self-efficacy for screening using FIT, or attitudes and confidence toward completing FIT testing [24-26,29,30,49,55]. For each respective construct, the corresponding items’ scores reported by participants were added together; construct total scores were used for analyses.

### Statistical Analysis

Sociodemographic characteristics and PHM constructs were summarized using descriptive statistics. The assessment details for each research question are discussed in the following four sections.

#### Research Question 1

RQ1 is as follows: To what extent do SD Latino adults use the internet to locate OHI? OHI seeking was characterized using descriptive statistics.

#### Research Question 2

RQ2 is as follows: How are self-reported levels of eHealth literacy described among SD Latino adults who seek OHI? eHealth literacy was characterized using descriptive statistics.

#### Research Question 3

RQ3 is as follows: What sociodemographic characteristics and PHM constructs are associated with OHI seeking by SD Latino adults? Univariate logistic regression analyses were conducted to examine potential sociodemographic and PHM constructs associated with OHI seeking. Data from all study participants (N=76) were available for this exploration. For these analyses, we coded responses to the item “Personally looked online for health information for self or others” as a binary outcome. Gender, insurance status, employment status, marriage status, age, annual income, educational attainment, perceived salience, perceived susceptibility, response efficacy, cancer worry, social influence, religious beliefs, and self-efficacy for screening with FIT were treated as independent variables, and whether participants sought OHI for themselves or others—yes or no response—was treated as the dependent variable.

#### Research Question 4

RQ4 is as follows: What sociodemographic characteristics and PHM constructs are preliminarily associated with eHealth literacy among SD Latino adults who seek OHI? Univariate analyses were conducted leveraging linear regressions to examine potential sociodemographic characteristics and PHM constructs associated with eHealth literacy (ie, the eHEALS score). The eHealth literacy outcome score was treated as a continuous outcome, ranging from 8 to 40. For this exploration, data were available only from those seeking OHI (22/76, 29%). Gender, insurance status, employment status, marriage status, age, annual income, educational attainment, perceived salience, perceived susceptibility, response efficacy, cancer worry, social influence, religious beliefs, and self-efficacy for screening with FIT were treated as independent variables, and the eHEALS score was treated as the dependent variable. Due to the sample size limitation, this exploration was underpowered; hence, the goal was to provide reasonably reliable estimates to guide the design of a future, larger, appropriately powered study.

#### General Analysis

Analyses were conducted using SAS (version 9.4 [TS1M6]; SAS Institute Inc). A *P* value of less than .05 was considered statistically significant. Of note, analyses are exploratory and not for definitive inferential interpretations.

## Results

### Sample Characteristics

Sociodemographic characteristics are described in Table 1 and in the main reported outcomes of Gwede et al [30]. In total, 67% (51/76) of participants were female. The mean age of the participants was 57.2 (SD 6.0) years (range 50-74). In total, 62% (47/76) of participants reported not completing high school. In addition, 41% (31/76) of participants reported being unemployed and having an annual income of less than US $10,000. Further, 75% (57/76) of participants lacked health insurance. In total, 93% (71/76) of participants reported being born outside of the United States. Among those born outside of the United States, a majority (49/71, 69%) reported Mexico as their country of birth. Further, among those born outside of the United States, the mean length of time reported living in the United States was 23.4 (SD 10.9) years (range 2-56).

**Table 1:** Summary of descriptive statistics.

Variables		Full sample (N=76)		OHI^a^ seekers (n=22)	
Age (years), mean (SD), range		57.2 (6.0), 50-74		56.6 (5.8), 50-69	
Years in the United States, mean (SD), range		23.4 (10.9), 2-56		19.4 (11.2), 2-56	
**Gender, n (%)**					
	Male	25 (33)			3 (4)
	Female	51 (67)			19 (25)
**Race, n (%)**					
	White	23 (30)			8 (11)
	Black	1 (1)			1 (1)
	Other or more than one race	52 (68)			13 (17)
**Marital status, n (%)**					
	Married or partnered	53 (70)			14 (18)
	Divorced or separated	10 (13)			5 (7)
	Widowed	6 (8)			3 (4)
	Never married or single	7 (9)			0 (0)
**Employment status, n (%)**					
	Employed or self-employed	40 (53)			16 (21)
	Unemployed	31 (41)			6 (8)
	Student	1 (1)			0 (0)
	Retired	3 (4)			0 (0)
**Education, n (%)**					
	Less than high school	47 (62)			3 (4)
	High school graduate	13 (17)			6 (8)
	Some college or technical school	7 (9)			5 (7)
	College graduate	7 (9)			6 (8)
	Graduate or professional (postcollege)	2 (3)			2 (3)
**Annual income (US $), n (%)**					
	<10,000	31 (41)			8 (11)
	10,000-25,000	29 (38)			10 (13)
	25,001-35,000	8 (11)			2 (3)
	35,001-75,000	2 (3)			1 (1)
	Don’t know or prefer not to answer	6 (8)			1 (1)
**Insurance status, n (%)**					
	No insurance	57 (75)			14 (18)
	Medicaid or Medicare	4 (5)			0 (0)
	County health insurance	8 (11)			3 (4)
	Private health insurance	7 (9)			5 (7)
**Country of birth, n (%)**					
	United States	5 (7)			2 (3)
	Other	71 (94)			20 (26)
**Self-reported country of birth (if that reported was other than the United States), n (%)**					
	Mexico	49 (64)	7 (9)		
	Colombia	6 (8)	4 (5)		
	Puerto Rico	5 (7)	2 (3)		
	Costa Rica	3 (4)	2 (3)		
	Dominican Republic	2 (3)	0 (0)		
	Peru	2 (3)	2 (3)		
	Chile	1 (1)	1 (1)		
	Cuba	1 (1)	1 (1)		
	El Salvador	1 (1)	0 (0)		
	Venezuela	1 (1)	1 (1)		
**Parents born outside the United States, including Puerto Rico, n (%)**					
	Yes	73 (96)	21 (28)		
	No	3 (4)	1 (1)		
**Personally sought OHI, n (%)**					
	Yes	22 (29)	22 (29)		
	No	54 (71)	0 (0)		
eHEALS^b^ score, mean (SD), range		N/A^c^	29.73 (6.6), 15-40		

^a^OHI: online health information.

^b^eHEALS: eHealth Literacy Scale; the total score ranged from 8 to 40; 22 out of 76 participants completed the scale.

^c^N/A: not applicable; this was not calculated for the full sample, since not all participants completed the scale.

### Main Findings

#### Extent to Which SD Latino Adults Use the Internet to Locate OHI (RQ1)

All participants (N=76) responded to the item assessing personal OHI seeking for themselves or others. In total, 71% (54/76) of participants reported not having personally sought OHI for themselves or others (Table 1), whereas the remaining 29% (22/76) of participants reported having personally looked for OHI information for themselves or others.

#### Variability in Self-reported Levels of eHealth Literacy Among SD Latino Adults (RQ2)

Assessment of eHealth literacy using the eHEALS was only completed for those who answered yes to having sought OHI for themselves or others (22/76, 29%). Of those individuals, the mean eHEALS score was 29.7 (SD 6.6; range 15-40). In total, 27% (6/22) of those seeking OHI had an eHEALS score of less than 26, indicating that these participants were in the low–eHealth literacy category.

**Figure 1:**
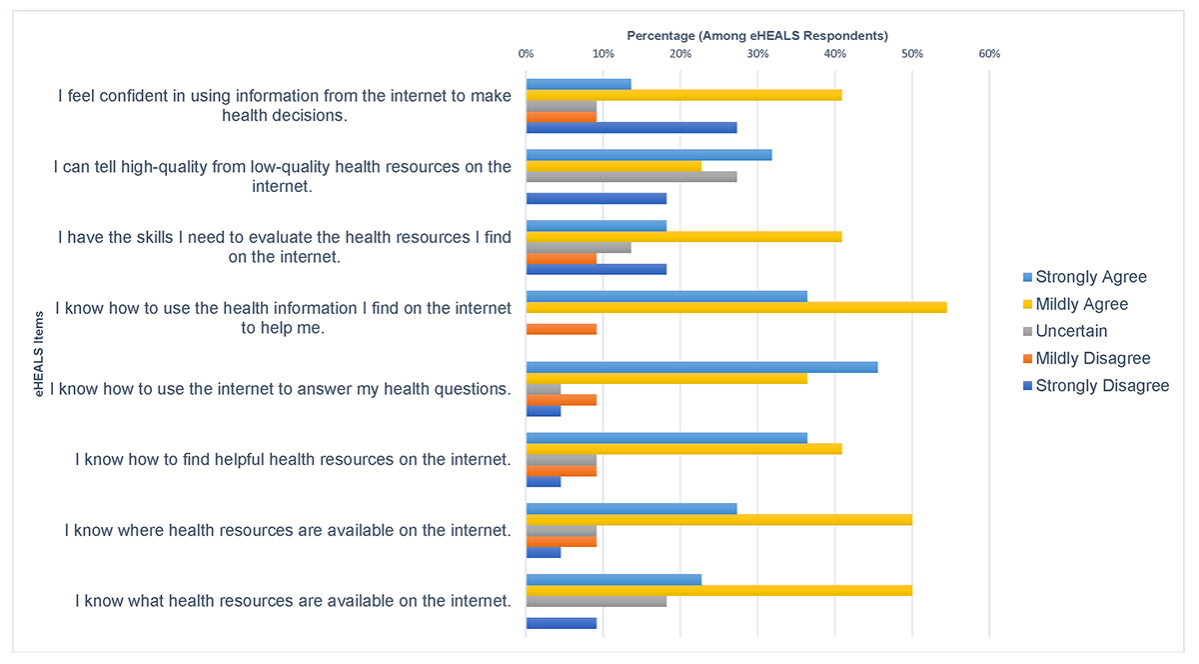
Responses to the eight-item eHEALS; 22 out of 76 respondents completed the scale, and percentages are out of 22. eHEALS: eHealth Literacy Scale.

Frequency of responses to the eight-item eHEALS—note that only 22 participants completed this scale, as they sought OHI—is reported in Figure 1. The item with the greatest degree of agreement was “I know how to use the health resources I find on the internet to help me,” with 91% (20/22) of eHEALS respondents self-reporting either mildly agree or strongly agree. The item with the least amount of agreement was “I feel confident in using information from the internet to make health decisions,” with 45% (10/22) of eHEALS respondents self-reporting being uncertain, mildly disagree, or strongly disagree.

#### Sociodemographics and PHM Constructs Associated With OHI Seeking by SD Latino Adults (RQ3)

Univariate logistic regression analyses on data from 76 participants were performed to examine preliminary associations between sociodemographic variables, PHM constructs, and the likelihood of seeking OHI. Only educational attainment was found to be significantly associated with OHI seeking (Table 2). Higher educational attainment was significantly associated with an increased likelihood of having sought OHI (odds ratio 17.4, 95% CI 2.0-150.7; *P*=.009). Employment status approached significance (*P*=.07). None of the PHM health beliefs were significantly associated with OHI. The logistic regression model was statistically significant (*χ*^2^_14_=60.3, *P*<.001). The model explained 81.9% (Nagelkerke *R*^2^) of the variance in OHI and correctly classified 90.0% of cases. Sensitivity was 85.7%, specificity was 91.8%, positive predictive value was 81.8%, and negative predictive value was 93.8%.

**Table 2:** Factors associated with seeking of online health information using univariate logistic regression analyses.

Covariate			Respondents (N=76), n (%)		Odds ratio (95% CI)		*P* value^a^
**Gender**							
	Female	51 (67)		170.05 (0.37 to >999.99)		.10	
	Male	25 (33)		Reference		N/A^b^	
**Insured**							
	Yes	19 (25)		59.19 (0.42 to >999.99)		.11	
	No	57 (75)		Reference		N/A	
**Employed**							
	Yes	40 (53)		>999.99 (0.57 to >999.99)		.07	
	No	35 (46)		Reference		N/A	
**Married**							
	Yes	53 (70)		62.01 (0.20 to >999.99)		.16	
	No	23 (30)		Reference		N/A	
Age			76 (100)		0.93 (0.64 to 1.35)		.68
Annual income			70 (92)		2.03 (0.50 to 8.29)		.32
Educational attainment			76 (100)		17.40 (2.01 to 150.72)		.009
Perceived salience			76 (100)		1.84 (0.45 to 7.44)		.39
Perceived susceptibility			76 (100)		0.99 (0.53 to 1.85)		.98
Response efficacy			76 (100)		2.33 (0.32 to 17.10)		.41
Cancer worry			76 (100)		1.18 (0.70 to 2.02)		.53
Social influence			76 (100)		1.07 (0.60 to 1.90)		.83
Religious beliefs			76 (100)		0.95 (0.74 to 1.22)		.68
Self-efficacy for screening with FIT^c^			76 (100)		0.96 (0.43 to 2.14)		.91

^a^*P*<.05 indicates statistical significance.

^b^N/A: not applicable.

^c^FIT: fecal immunochemical test.

#### Sociodemographics and PHM Constructs Associated With the eHealth Literacy of SD Latino Adults (RQ4)

Univariate analyses using linear regressions were completed to examine preliminary associations between eHEALS scores, sociodemographic variables, and PHM constructs. Higher self-efficacy for screening with FIT was significantly associated with higher eHEALS scores (ie, eHealth literacy; *β*=1.20, 95% CI 0.14-2.26; *P*=.02). Table 3 reports the regression coefficients and standard errors. The best-fit model was significantly associated with eHEALS score (*F*_1,20_=5.53, *P*=.02; adjusted *R*^2^=0.18).

**Table 3:** Results from univariate analyses using linear regressions for two models.

Factors associated with the eHEALS^a^		*β* coefficient (SE)	Standardized *β*	*t* test (*df*=1)	*P* value^b^	
**Model 1**						
	Constant	4.22 (56.50)	0	0.07	.94	
	Gender	–3.19 (10.53)	–0.18	–0.30	.77	
	Insured	1.12 (7.49)	0.09	0.15	.88	
	Employed	–1.71 (9.88)	–0.12	–0.17	.87	
	Married	3.51 (7.86)	0.26	0.45	.67	
	Age	0.17 (0.45)	0.16	0.37	.72	
	Annual income	–0.60 (5.04)	–0.08	–0.12	.91	
	Educational attainment	–0.19 (2.99)	–0.04	–0.06	.95	
	Perceived salience	–0.57 (2.88)	–0.11	–0.20	.84	
	Perceived susceptibility	–0.82 (1.18)	–0.32	–0.69	.51	
	Response efficacy	–0.46 (2.36)	–0.08	–0.19	.85	
	Cancer worry	0.09 (1.66)	0.04	0.06	.96	
	Social influence	–0.10 (1.30)	–0.05	–0.08	.94	
	Religious beliefs	–0.33 (1.00)	–0.25	–0.34	.75	
	Self-efficacy for screening with FIT^c^	1.58 (1.17)	0.64	1.35	.22	
**Model 2**						
	Constant	–6.41 (14.51)	0	–0.44	.66	
	Self-efficacy for screening with FIT	1.20 (0.51)	0.47	2.35	.02	

^a^eHEALS: eHealth Literacy Score.

^b^*P*<.05 indicates statistical significance.

^c^FIT: fecal immunochemical test.

## Discussion

### Principal Findings

OHI has become more routine in health care interactions and health education for patients and caregivers [3-5]. Yet, greater emphasis on eHealth may disproportionately affect groups with limited eHealth literacy. Healthy People 2030 continues the national effort initiated in 2010 in recognition of the importance of HC and HIT to support better communication, care, and outcomes toward achieving health equity [56]. There is a dearth of information available in the literature that communicates specifically on factors (eg, sociodemographic characteristics and health beliefs) associated with OHI seeking and eHealth literacy among SD Latino adults.

In this study, nearly three-quarters (54/76, 71%) of participants reported not having personally sought OHI for themselves or others. Among those for whom eHealth literacy was assessed (22/76, 29%), nearly one-third (6/22, 27%) had an eHEALS score of less than 26, indicating self-perceived low eHealth literacy. In this study, preliminary findings suggest that higher educational attainment was associated with an increased likelihood of having sought OHI. Although further research to confirm directionality is necessary, this association between higher educational attainment and seeking OHI among SD Latino adults is consistent with prior findings on OHI behaviors conducted among English-speaking populations [57,58]. Specifically, Jacobs et al [58] highlight that a heavy reliance on e-technologies for disseminating health information may increase the likelihood of further perpetuating health disparities; they suggest a need for interventions and efforts focused on developing training and services to boost internet self-efficacy tailored to patients’ learning styles and their cultural and demographic characteristics to reduce this digital disparity.

This study was conducted among SD Latino adults of whom the majority were born outside of the United States, which is acutely different from studies conducted among participants preferring the English language [57,58]. Our efforts are preliminary and, thus, additional research is needed to explore the relationships between educational attainment and OHI seeking among various groups who prefer non-English languages and groups newly arrived in the United States. Of interest, in our exploration of factors associated with the act of OHI seeking, there was a high proportion of individuals who had not sought OHI (54/76, 71%). This lack of OHI seeking may disproportionately affect SD Latino adults, as reliance on eHealth resources continues to gain emphasis in the United States [3-5] and globally [59]. Indeed, our efforts are cautiously interpreted, yet our data signal a timely opportunity to examine the reasons preventing OHI-seeking behaviors among SD Latino adults aged 50 to 75 years, as well as the potential for intervention research. Thus, in recognizing this distinction, considerations for culture and language are a fertile area of future research examining reasons for the lack of OHI seeking (eg, learning styles and preferences, availability and access to Spanish-language computer resources, knowledge, and technological skills, such as search strategies, among others). In considering culture, previous studies exploring OHI behaviors suggested that younger, English-dominant Latino and Hispanic generations may be OHI brokers for older generations of SD Latino adults [4,35,60]. Therefore, whether OHI seeking could be facilitated by intergenerational co-learning approaches is an avenue for future exploration. In a study examining digital health disparities among ethnically diverse older adults who prefer the English language [61], researchers suggested important steps to close this digital divide, including cultural adaptation based on preferences for receiving health information. Research has found that personal instruction through the process of internet use and assistance with using new digital devices helps older adults to adopt a daily habit of internet use [62]. However, whether these outcomes may be replicated among SD Latino adults aged 50 to 75 years remains to be examined. Further still, the literature communicates that smartphone ownership offers no statistically significant difference relevant to race or ethnicity [63] and is the primary source of internet use among all Latino adults [63]. Thus, considerations for examining the potential of leveraging smartphones for interventions on promoting OHI seeking among SD Latino adults is worth noting. Moreover, in addition to reaching underserved populations in health centers [64], community libraries offer another potential avenue for engaging with diverse community members [65].

In our study, exploratory findings suggest that higher eHealth literacy was preliminarily associated with higher self-efficacy for FIT screening (*β*=1.20, 95% CI 0.14-2.26; *P*=.02) among SD Latino adults. This preliminary finding suggests that further investigation is warranted. Future in-depth examinations are necessary to confirm directionality of association. Prior to this study, the literature did not communicate specifically regarding findings that have examined eHealth literacy and self-efficacy for CRC screening. Efforts by Park et al [66] conveyed an association between higher levels of eHealth literacy and greater confidence in seeking online cancer information. Yet, the study was completed among English-language participants; importantly, the “confidence” examined by Park et al [66] differs from our use of “self-efficacy” that we occupy via the PHM.

This preliminary study communicates promising results suggesting that increased eHealth literacy may be associated with increased self-efficacy for CRC screening with FIT. While this study’s results must be replicated in a larger sample, these findings are encouraging and set an important small guiding step in suggesting that eHealth literacy skills development might benefit self-efficacy of CRC screening. Future appropriately powered research is necessary to examine whether eHealth literacy training and building self-efficacy for CRC screening may ultimately impact CRC screening uptake.

### Limitations and Strengths

This study has several limitations and strengths to acknowledge. First, the study was conducted in the context of a small pilot trial, underpowered for inferential analyses. Thus, our findings should be interpreted cautiously and call for further study in larger samples of SD Latino individuals. Second, this study did not assess access to the internet nor barriers to OHI seeking, limiting potential findings. Third, Latino ethnicity is shared among many Americans from various backgrounds and, most certainly, does not comprise a homogenous group. Characteristics of Latino adults vary by region. In this study, participants communicated their Latino ethnicity, and most were of Mexican heritage, thus potentially limiting generalizability. Yet, the sample representing 10 different Latin American countries offers preliminary evidence of feasibility for recruiting Latino adults from diverse backgrounds into future studies.

### Implications

There are several implications to consider and acknowledge. First, to reach the Healthy People 2030 HC and HIT objectives, it is necessary to expand research efforts to highlight and address barriers and leverage facilitators to OHI behaviors; it is also necessary to increase access to the internet. Second, future intervention research to promote eHealth literacy should examine and consider how cultural background and language affect access, health care use, successful use of eHealth resources, and behavior change. Finally, this study provided preliminary findings that suggested a need for pursuing further research on promoting OHI-seeking behaviors and eHealth literacy training that may impact CRC self-efficacy and, ultimately, CRC screening behaviors. Nevertheless, larger research studies are needed to corroborate these findings before clear implications for practice can be reached.

### Conclusions

This preliminary study adds to an extremely small evidence base and is the first to communicate findings on the assessment and analyses of OHI seeking and eHealth literacy in the context of the PHM among SD Latino adults. A high proportion of SD Latino participants in our study have not sought OHI for themselves or others, thus limiting their access to beneficial resources. In light of the growing use and reliance on technologies in health care, factors preventing OHI seeking remain to be further examined. In addition, there is a need for resources to improve eHealth literacy among SD Latino adults. Our study communicates preliminary evidence that higher eHealth literacy is occurring among those with higher self-efficacy for CRC screening. Appropriately powered research in the future is warranted to further examine this preliminary finding. Additionally, the next logical step that future research should examine is whether eHealth literacy training and building self-efficacy for CRC screening could increase the uptake of CRC screening.

## Abbreviations

CRC: colorectal cancer
eHEALS: eHealth Literacy Scale
FIT: fecal immunochemical test
FQHC: Federally Qualified Health Center
HC: health communication
HIT: health information technology
HRQOL: health-related quality of life
Latino CARES: Latino Colorectal Cancer Awareness, Research, Education, and Screening
NCI: National Cancer Institute
OHI: online health information
PHM: Preventive Health Model
PI: principal investigator
RQ1: research question 1
RQ2: research question 2
RQ3: research question 3
RQ4: research question 4
SD: Spanish-dominant
